# Targeted P2X7/NLRP3 signaling pathway against inflammation, apoptosis, and pyroptosis of retinal endothelial cells in diabetic retinopathy

**DOI:** 10.1038/s41419-022-04786-w

**Published:** 2022-04-12

**Authors:** Hui Kong, Hongran Zhao, Tianran Chen, Yanling Song, Yan Cui

**Affiliations:** 1 Shandong University of Traditional Chinese Medicine, Jinan, Shandong Province China; 2 Department of Ophthalmology, Qianfoshan Hospital of Shandong First Medical University, Jinan, Shandong Province China; 3 NHC Key Laboratory of Otorhinolaryngology, Qilu Hospital of Shandong University, Jinan, Shandong Province China; 4 Department of Ophthalmology, Qilu Hospital of Shandong University, Shandong University, Jinan, Shandong Province China; 5 Shandong University, Jinan, Shandong Province China

**Keywords:** Apoptosis, Cell death

## Abstract

Retinal endothelial cells (RECs) are the primary target cells for diabetes-induced vascular damage. The P2X7/NLRP3 pathway plays an essential role in amplifying inflammation via an ATP feedback loop, promoting the inflammatory response, pyroptosis, and apoptosis of RECs in the early stages of diabetic retinopathy induced by hyperglycemia and inflammation. 3TC, a type of nucleoside reverse transcriptase inhibitor, is effective against inflammation, as it can targeting formation of the P2X7 large pore formation. Hence, our aim was to evaluated the anti-inflammatory effects and potential mechanisms of action of 3TC in vitro in retinal microvascular endothelial cells treated with high-glucose (HG) and lipopolysaccharide (LPS), as well as in vivo in the retinas of C57BL/6J male mice with streptozotocin-induced diabetes. The expression of inflammasome-related proteins P2X7 and NLRP3, and apoptosis in the retinas of 3TC-treated diabetic mice were compared to those of untreated diabetic mice. Furthermore, the anti-inflammatory, anti-apoptotic, and anti-pyroptotic effects of 3TC were evaluated in vitro in cultured mice retinal endothelial cells. Co-application of HG and LPS significantly increased the secretion of IL-6, IL-1β, and TNF-α, and ATP levels, whereas 3TC decreased cell inflammation, apoptosis, and pyroptosis. Inhibition of P2X7R and NLRP3 inflammasome activation decreased NLRP3 inflammasome-mediated injury. 3TC prevented cytokine and ATP release following co-application of HG and LPS/BzATP. Our findings provide new insights regarding the mechanisms of action of 3TC in diabetic environment-induced retinal injury, including apoptosis and pyroptosis.

## Introduction

Diabetic retinopathy (DR) is the most common complication associated with diabetes mellitus, which leads to severe visual impairment and blindness. Retinal endothelial cells (RECs) are one of the major cell types involved in DR [1]. In the early stages of DR, hypoperfusion may contribute to low-grade chronic vascular inflammation in retinal capillaries (leukostasis) and progressive and irreversible hypoxia, eventually leading to microaneurysm and formation of acellular capillary [2]. Although neovascularization causes severe vision loss only in the proliferative phase of late DR, macular edema caused by vascular leakage can occur at any stage of DR and impair visual acuity [3]. Although pericyte loss is an early characteristic change in DR, local vascular leakage occurs earlier in experimental diabetic mice, even before pericyte loss [4]. Alterations in the retinal endothelium play a crucial role in developing retinal diseases and are a hallmark of DR.

Hyperglycemia and inflammation are the main factors affecting DR pathophysiology [5, 6]. The inflammatory response caused by metabolic changes in diabetes damages the retinal neurovascular unit (RNUT), leading to gradual and progressive neuropathy [7, 8]. Neuroinflammation is actively related to P2X7R activation by risk-associated molecular patterns (DAMPs), the principal among which is extracellular ATP [9]. P2X7 receptors are expressed on many types of retinal cells, including RECs, and ATP is the only physiological agonist of P2X7. High glucose (HG) induces retinal pericyte cell death by activating P2X7R, and the ATP released from dying cells acts as a “danger signal”, further amplifying the inflammatory signal initiated by the glucose insult [10]. Human retinal endothelial cells (hRECs) exposed to HG expressed high P2X7R levels, further, an effect amplified by treatment with 2′-3′-O-(4-benzoyl-benzoyl) ATP (BzATP), a selective P2X7R agonist [11]. The CD40–ATP–P2X7 signaling pathway mediates cell-to-cell crosstalk in RNUT, and promotes inflammation and programmed cell death of endothelial cells, which is critical in the development of capillary degeneration and retinal ischemia [12]. P2X7R is involved in diabetic pathogenesis by triggering inflammasome activation and releasing inflammatory cytokines [13, 14]. P2X7R stimulation activates the NLR family protein 3 (NLRP3) inflammasome and triggers interleukin (IL)-1β maturation and secretion [15, 16].

Lipopolysaccharide (LPS) is another factor responsible for insulin resistance [17]. In DR, complex microbial systems result in high LPS levels in the serum [18]. Low-grade increase in plasma LPS level, termed “metabolic endotoxemia”, is a well-known feature of type 2 diabetes mellitus [19, 20]. Some studies have indicated that cytoplasmic LPS induces the opening of large P2X7-associated pores via ATP-mediated P2X7 signaling, and that caspase-11 activation engages the pannexin1 (PANX1) channel to induce NLRP3 activation [21].

The NLRP3 inflammasome comprises NLRP3, the adaptor protein ASC, and the effector protein Caspase-1. Upon activation, the NLRP3 inflammasome mediates caspase-1 activation, cleaving proIL-1β and proIL-18 into their active forms [22]. Activation of the NLRP3 inflammasome is critical for the progression of pro-inflammatory events in retinopathy. MCC950 decreases the expression of NLRP3 inflammasome in the retina of rats with DR and in hRECs cultured in the presence of high glucose levels in vitro [23]. P2X7R and NLRP3 inflammasome interact and co-localize in the cytoplasm. The NLRP3 inflammasome senses P2X7 receptor activation and transduces it into pro-inflammatory signals [24]. Mainly due to K^+^ efflux, Calcium dysregulation, and glutamine effluxs. P2X7R inhibition indirectly reduces NLRP3 inflammasome activation in retinal microglia [24].

In this study, we selectively activated P2X7R with BzATP and stimulated NLRP3 with LPS in mouse RECs (mRECs) cultured in the presence of HG to evaluate the anti-inflammatory anti-inflammatory, anti-apoptotic and anti-pyroptotic effects of 3TC on regulation of the P2X7/ NLRP3 inflammasome pathway.

We also investigated the synergistic effects of LPS and HG on mREC apoptosis to understand the role of P2X7/NLRP3 in early DR. Furthermore, we verified the effect of blocking P2X7R on LPS and HG-induced mREC apoptosis both in vitro and in vivo.

## Results

### P2X7 and NLRP3 expression levels were elevated in the diabetic retina

**Fig. 1:**
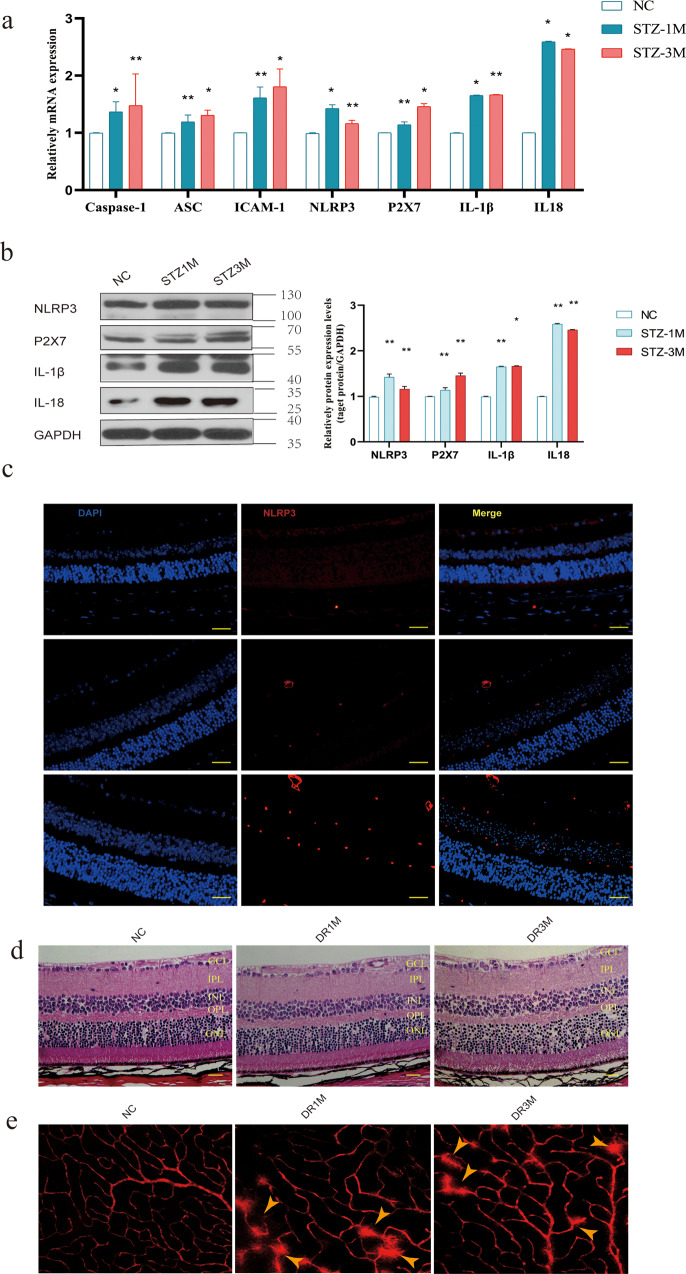
P2X7/NLRP3 signaling pathway activity arises in STZ-induced diabetic mice. **a** The relative mRNA expression of caspase 1, ASC, Icam1, P2X7, NLRP3, IL-1β, and IL-18 at different time points (4 and 12 weeks after STZ induction). **b** The relative protein levels of P2X7, NLRP3, IL-1β, and IL-18 in the retinal tissue of mice at the 4th and 12th weeks. **c** Immunofluorescence analysis and nuclear staining of retinal tissue sections from each group of mice with anti-NLRP3 antibody (red) and DAPI (blue), from top to bottom, normal control; diabetic mice (induced with STZ for one month); diabetic mice (induced with STZ for three months). **d** Representative photomicrographs showing hematoxylin-eosin (HE) staining of retinal sections. Scale bar = 50 μm. **e** Mice were infused with EB dye for 10 min detected the fluorescence signal of the flat-mounted retinas using a fluorescence microscope. Data are expressed as the mean ± SD (*n* = 3). *P < 0.05, **P < 0.01, ***P < 0.001 vs. the control group.

Inflammation plays an essential role in DR, an inflammatory cascade occurring in RNUT. The retinal microglia secrete inflammatory factors and induce REC death. After streptozotocin (STZ) treatment for 4 and 12 weeks, P2X7 mRNA expression was significantly higher in the retinal tissues of DR mice compared with the control mice. In addition, the mRNA levels of Casp1, ASC, NLRP3, inflammatory cytokines IL-1β and IL-18, and the intercellular adhesion factor ICAM-1 were increased in diabetic mice (Fig. 1a). To further validate the activation of the P2X7/NLRP3 inflammasome pathway in DR, the expression levels of the NLRP3 inflammasome, P2X7, IL-18 and IL-1β were assayed using western blotting (Fig. 1b). After STZ treatment, the NLRP3, IL-18, and IL-1β levels increased significantly at 4 and 12 weeks.

Further immunofluorescence analysis indicated that NLRP3 expression in DR mice was considerably higher than that in control mice (Fig. 1c). At 1 and 3 months of DR, we tested the structural morphology of the retinal cells. Hematoxylin-eosin (HE) staining did not show any significant decrease in retinal thickness due to diabetes (Fig. 1d). DR alters the retinal microvasculature, increases vascular permeability, and induces pathological intraocular proliferation of retinal vessels. The integrity of the blood-retinal barrier was determined by measuring EB extravasation in mouse retinas. As shown in Fig. 1e, retinal vascular leakage significantly increased in diabetic mice.

### Targeted inhibition of the P2X7/NLRP3 signaling pathway alleviated inflammatory activity and suppressed apoptosis

**Fig. 2:**
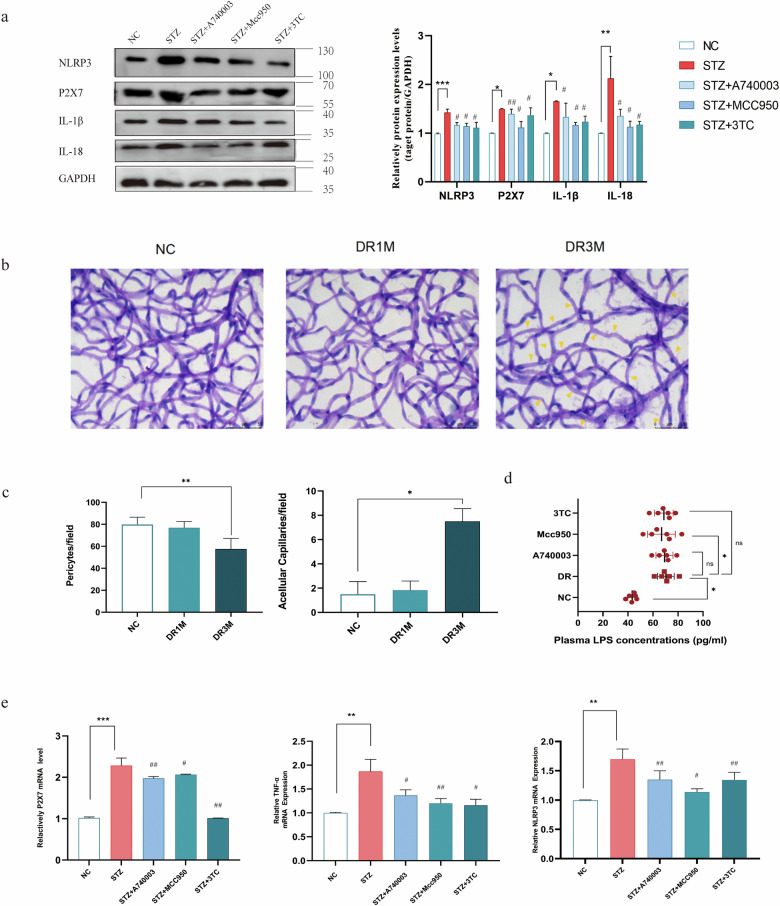
Inhibitions of the P2X7/NLRP3 signaling pathway activity in STZ-induced diabetic mice. **a** The relative protein expression of P2X7, NLRP3, IL-1β, and IL-18 in the retinal tissue of mice at the 12th week, inhibition of P2X7 can reduce the expression of P2X7, NLRP3, and the downstream inflammatory factors IL-1β, IL-18. **b**, **c** Trypsin digestion of the retina for detecting the number of pericytes and acellular capillaries after one and three months; yellow arrows indicate acellular capillaries. Pericytes and acellular capillaries were quantified in 10 random fields per retina and averaged (*n* = 6). Scale bar: 50 μm. **d** The plasma LPS concentrations in normal and STZ-induced 3-month mice groups. **e** The relative mRNA expression of TNF-α, P2X7, and NLRP3 in the retinal tissue of mice at the 12th week. Data are expressed as the means ± SD (*n* = 3). *P < 0.05, **P < 0.01, ***P < 0.001 vs. the control group.#*P* < 0.05, ##*P* < 0.01, vs. the DR group or STZ group.

A74003 and MCC950 are potent and specific inhibitors of P2X7 and NLRP3, respectively. 3TC, a type of multiple nucleoside reverse transcriptase inhibitor (NRTI), has been widely used to treat HIV infection. Recent studies have shown the intrinsic anti-inflammatory activity of 3TC, which targets P2X7 and the NLRP3 inflammasome pathway [25]. Intravitreous injection of 3TC can suppress laser-induced choroidal neovascularization in mice and downregulated VEGF-A via P2X7 [26]. Western blotting confirmed elevation of NLRP3, P2X7, IL-1β, and IL-18 protein expression in retinas of mice with DR, compared to those in retinas of control mice. Moreover, according to the WB analysis, the NLRP3, P2X7, IL-18, and IL-1β protein levels in the retinas of mice in the treatment groups demonstrated similar trends, indicating that 3TC reduced the inflammatory activity induced by hyperglycemia (Fig. 2a). Results of the retinal trypsin digestion assay indicated that hyperglycemia resulted in pericyte loss and acellular capillary formation. (Fig. 2b). Retinal capillaries were not significantly decellular after 4 weeks of STZ induction; however, decellularized capillary tissue increased at 12 weeks (Fig. 2c). After administration of A740003, MCC950, and 3TC, the mRNA expression of NLPR3 and TNF-α decreased compared with those in the DR group. P2X7 mRNA expression decreased after A740003 and 3TC inhibition (Fig. 2e). Concurrently, the number of decellularized capillaries decreased in the 3TC and A740003 groups. We examined plasma LPS levels in the circulating blood of mice and found plasma LPS levels increased in DR mice (Fig. 2d), consistent with the results of a previous study [27]. 3TC and A740003 did not reduce plasma LPS levels.

### High glucose, inflammatory cytokines, and LPS stimulated the production and secretion of inflammatory factors via the P2X7/NLRP3 signaling pathway

**Fig. 3:**
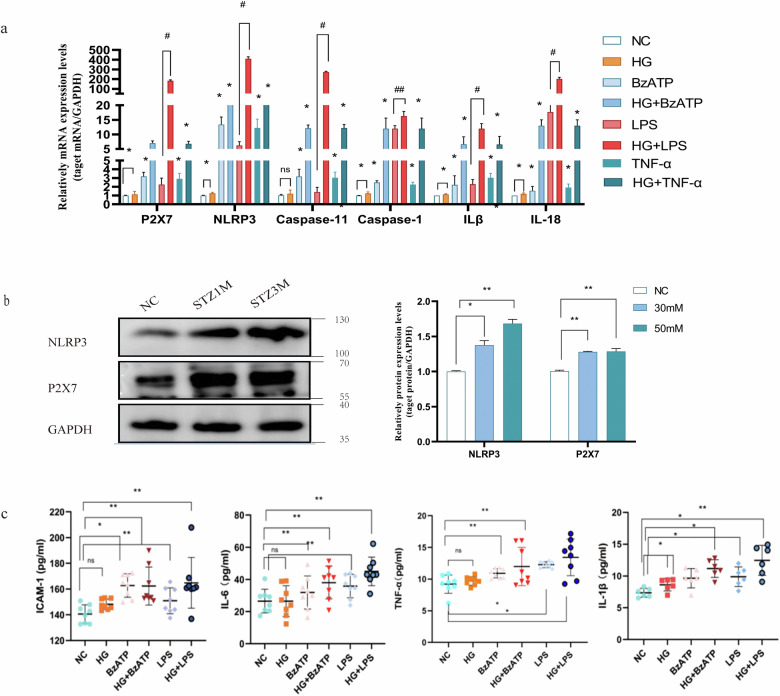
Expression of P2X7 and NLRP3 inflammasome and downstream inflammatory factors in mREC cultured in the presence of different glucose concentrations. **a** The relative mRNA expression of P2X7, NLRP3, Caspase 11, Caspase 1, IL-1β, and IL-18 under different conditions. **b** The relative protein expression levels of P2X7 and NLRP3 inflammasome under different conditions (5.5, 30, 50 mM D- glucose for 48 h. **c** The expression of TNF-α, IL-1β, Icam1, and IL-6 in cell culture medium supernatant under different conditions after 48 h was detected using customized ELISA kits. Cells treated with normal concentration of glucose (5.5 mM) were used as the control. Data are expressed as the mean ± SD (*n* = 3). *P < 0.05, **P < 0.01, ***P < 0.001 vs. the control group. #*P* < 0.05, ##*P* < 0.01, vs. the LPS group.

**Fig. 4:**
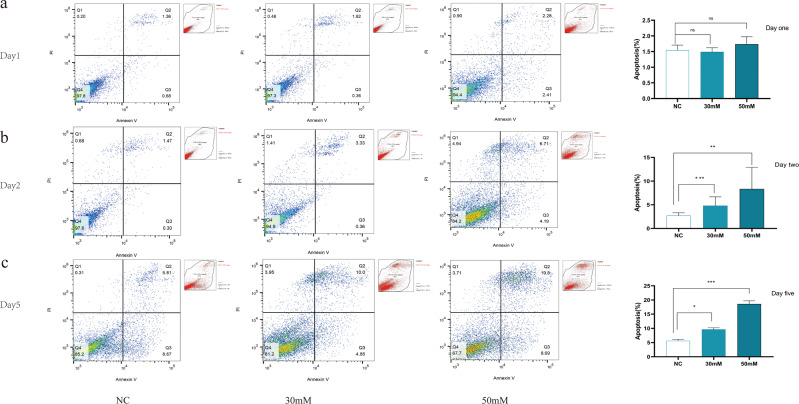
Combination of LPS and HG increased apoptosis and pyroptosis rates in mRECs. After treatment with 5.5 mM, 30 mM and 50 mM D glucose for different durations (24 h, 48 hand 5 days), cell apoptosis was measured using flow cytometry. **a** Cell apoptosis in the presence of 5.5 mM D- glucose, **b** Cell apoptosis in the presence of 30 mM D- glucose, **c** Cell apoptosis in the presence of 50 mM D- glucose, Data are expressed as the mean ± SD (*n* = 3). *P < 0.05, **P < 0.01, ***P < 0.001 vs. the control group.

The incidence of REC death increases in diabetes patients and animals with experimental diabetes or galactosemia [20]. However, conflicting results have been obtained in vitro studies. Some have shown reduction in cell viability and increase in apoptosis in RECs upon exposure to HG [28–31]. In other studies, hyperglycemia did not increase REC apoptosis [32]. but promoted cell proliferation [33–35]. Intermittent hyperglycemia had a higher proliferative effect on hRECs than continuous hyperglycemia. The effect of hyperglycemia on macrovascular and microvascular endothelial cells differed [36]. These results suggested that the response of RECs to HG may be complex, involving both direct and indirect effects, which may be affected by differences in species, cell preparation, and culture conditions. Our findings have shown that the transcription of P2X7, NLRP3, Casp 11, Casp 1, IL-1β, and IL-18 increased after HG/BzATP/TNF-α/LPS treatments (Fig. 3a). P2X7 and NLRP3 protein expression increased in RECs cultured in medium containing 30 mM and 50 mM glucose (Fig. 3b). HG induced P2X7R expression and release of the pro-inflammatory IL- 1β in hRECs [11]. However, the elevation of cytokines levels in response to HG stimulation alone was not significant. Analysis of the secretion of IL-6, ICAM-1, TNF-α, and IL-1β under basal conditions and in response to HG, LPS/BzATP, and co-application of HG and LPS/BzATP showed that administration of LPS/BzATP alone, but not HG alone, induced IL-6 release. However, compared to the basal levels, co-application of HG and LPS increased the levels of secreted IL-6 and ICAM-1 (Fig. 3c). There was no significant change in the apoptotic rate of cells cultured with different glucose concentrations for 24 h (Fig. 4a). The apoptotic rate of cells increased after 2 and 5 days in the presence of 30 and 50 mM glucose concentrations and reached 10.0% and 19.8% after 5 days, respectively (Fig. 4b, c). Notably, 25.5 mM glucose decreased the apoptotic rate more than 5.5 mM glucose, indicative of low-glycemic deprivation death. In subsequen experiments, 30 mM D-glucose was used for HG stimulation.

### Combination of LPS and HG induced opening of the large P2X7-associated pore and activation of NLRP3

**Fig. 5:**
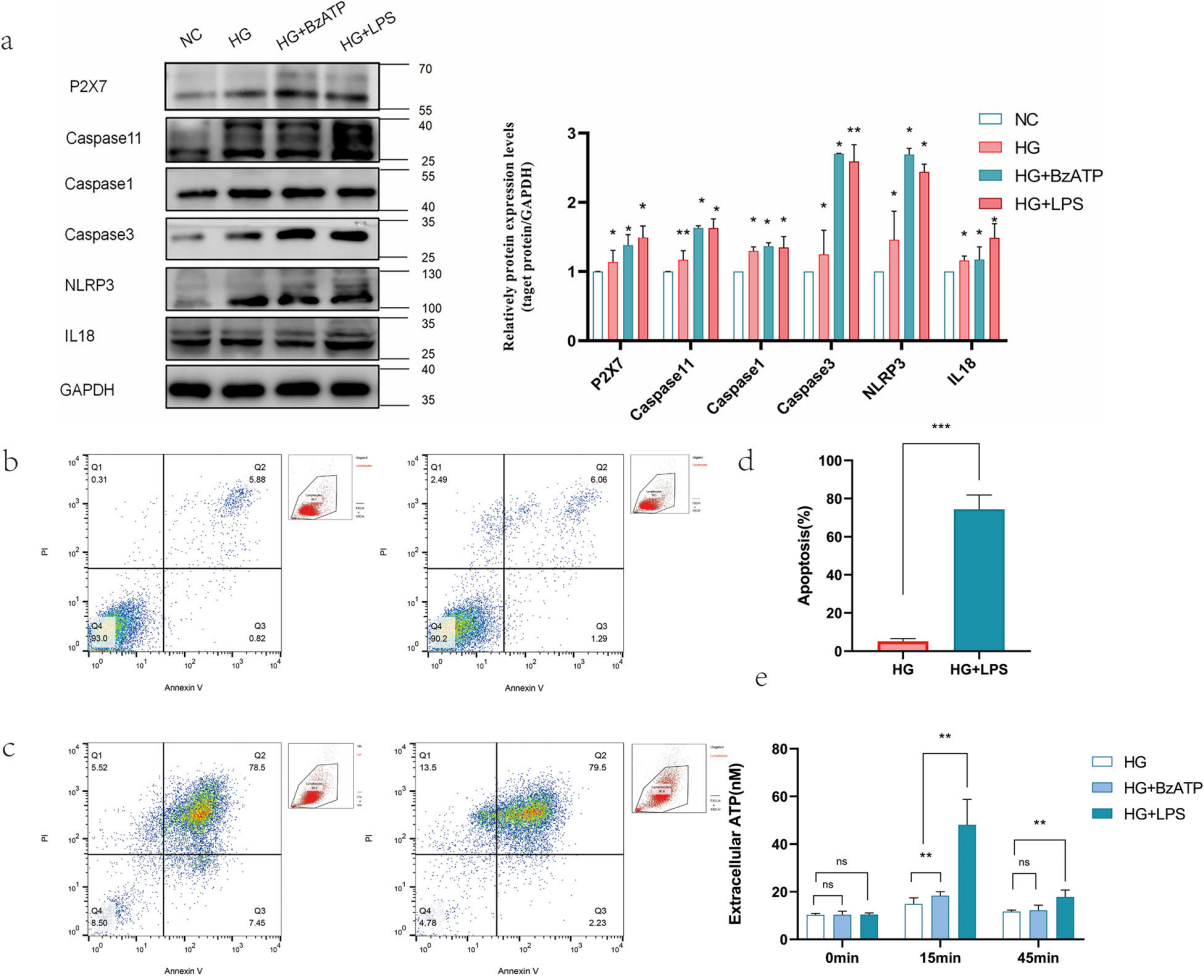
Combination of LPS and HG increased apoptosis and pyroptosis rates in mRECs by enhancing the P2X7/NLRP3 inflammasome signaling pathway. **a** Measured The protein expression levels of P2X7, NLRP3 inflammasome, Caspase 1, Caspase 11, Caspase 3, and IL-18 under different conditions (5.5, 30 mM D- glucose, 30 mM D- glucose + 100 µM BzATP, 30 mM D-glucose + 20 ng/ml LPS for 48 h using western blotting. **b**–**d** mRECs were cultured in HG (30 mM D-glucose) and HG + LPS (30 mM D-glucose combined + 20 ng/ml LPS) and apoptosis was assessed. **e** The extracellular ATP in mRECs was measured using an ATP bioluminescence assay kit at 15 and 45 min after stimulation of HG with or without LPS. Data are expressed as the mean ± SD (*n* = 3). *P < 0.05, *P < 0.01, ***P < 0.001 vs. the control group.

**Fig. 6:**
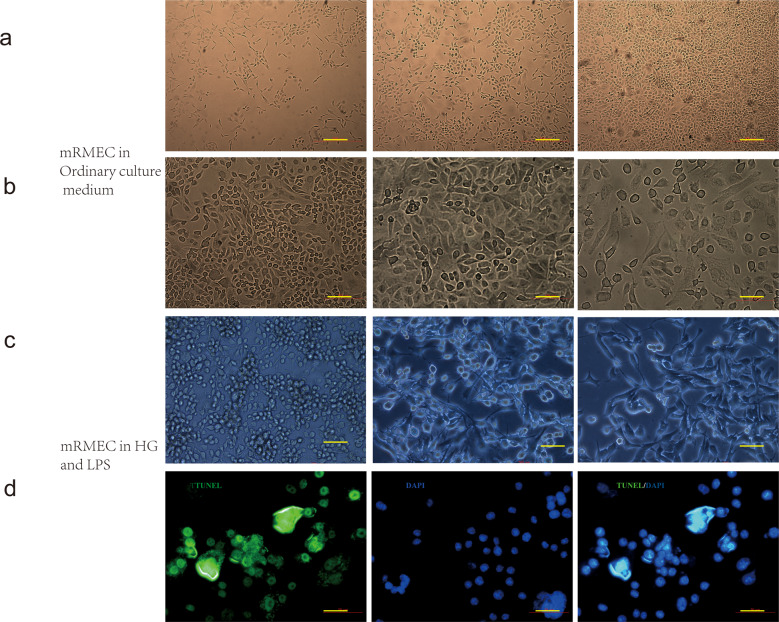
Morphological changes of cells after 7 days of incubation in high glucose medium with LPS. **a** mRECs cultured in ordinary culture medium (HyClone, DMEM), bar: 200 µm. **b** mRECs cultured in ordinary culture medium (HyClone, DMEM), bar:50 µm. **c** mRECs cultured in high glucose medium with LPS(20 ng/ml), bar:50 µm. **d** TUNEL (green fluorescence)staining in high glucose medium with LPS(20 ng/ml), bar:50 µm.

In diabetes patients, many pathogenic bacteria and their metabolites can enter the circulation via the intestine because of impairment of intestinal barriers and increase in permeability, which may result in systemic effects, including chronic low-grade inflammation [34]. We first investigated whether LPS, a metabolite of the intestinal flora, activated the P2X7/NLRP3 pathway and found that the combination of LPS and HG culture, induced transcription of P2X7 and NLRP3 in mRECs, which was even higher than that observed after treatment with BzATP, the direct agonist of P2X7 (Fig. 3a). The protein levels of caspase 11, caspase 3, caspase 1, and IL-18 increased (Fig. 5a). Addition of LPS under high glucose condition increased the apoptotic rate of mRECs (Fig. 5b, c, d); mRNA of IL-18, IL-1β, Casp 1, and cytokines in cell supernatant were higher than those observed with the combination of HG and BzATP (Fig. 3a, c). We hypothesized that LPS combined with HG culture, activated some ATP channels and set a self-feedback loop amplification loop of ATP secretion. The results of extracellular ATP measurement supported this hypothesis (Fig. 5e). Pannexin 1 and connexin are both involved in ATP release [37] and extracellular ATP. K^+^ efflux [38] and glutathione efflux [39] are common events occurring upstream of NLRP3 inflammasome activations. ATP is the only physiological agonist of P2X7. Under transient stimulation with ATP, the P2X7 receptor cation channels open, leading to efflux of K^+^ and influx of Na^+^ and Ca^2+^. Under continuous stimulation with ATP, the P2X7 receptor forms a non-selective membrane pore, allowing entry of substances with relative molecular masses of up to 900 kDa inside the cell, leading to cell death [40]. However, the channel-to-pore transition mechanism remains unclear [14]. The P2X7R macropore may also be opened by agonists acting on the cytoplasmic side. In macrophages, LPS induces P2X7R activation and ATP release [41]. Intracytoplasmic LPS lowers the threshold of P2X7R activation, thus sensitizing this receptor to ambient ATP concentrations [42]. LPS-induced P2X7 and NLRP3-associated GSDMD can form macropores to mediate pyroptosis. The cell morphology changed after 7 days of incubation in high glucose medium with LPS (Fig. 6a–c), and the cells were positive for TUNEL staining (Fig. 6d).

### Inhibition of P2X7R attenuated apoptosis and pyroptosis of mice retinal vascular endothelial cells in vitro

**Fig. 7:**
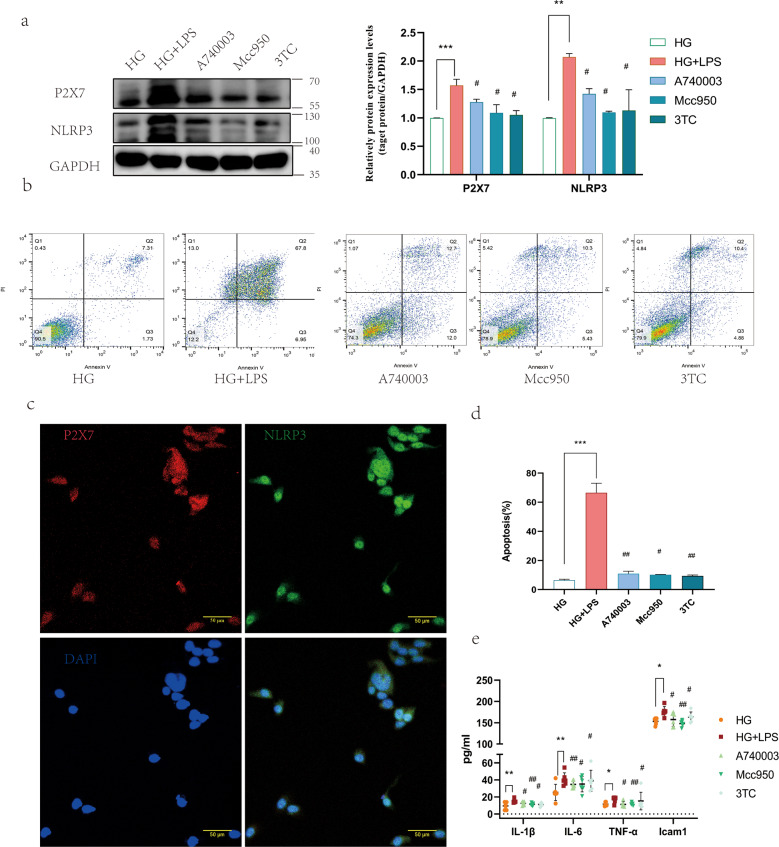
Inhibition of P2X7 alleviated LPS-induced apoptosis and pyroptosis in mRECs by attenuating the activation of P2X7 and NLRP3 inflammasome. **a** P2X7 and NLRP3 inflammasome protein levels. **b**, **d** Cell apoptosis was measured using flow cytometry. **c** Immunofluorescence co-staining on cells co-stimulated with high glucose and LPS for 7 days, P2X7(red fluorescence), NLRP3(green fluorescence), DAPI(blue fluorescence) **e** Secretion of IL-6, ICAM-1, TNF-α, and IL-1β was evaluated in cell culture medium under different conditions for 48 h using customized ELISA kits. Results are expressed as mean ± SD; Statistical analyses were performed using one-way ANOVA with Tukey’s multiple comparison’s test; *n* = 6; *t* = 48 h. Data are expressed as the means ± SD (*n* = 3). *P < 0.05, *P < 0.01, ***P < 0.001 vs. the control group. #*P* < 0.05, ##*P* < 0.01, vs. the HG + LPS group.

NRTIs are mainstay therapeutics for HIV infection. Recent studies have provided evidence that NRTIs can inhibit P2X7-mediated NLRP3 inflammasome activation independent of reverse transcriptase inhibition [21]. In addition, NRTIs act as promising anti-inflammation therapeutics by targeting P2X7-dependent large pore formation [26]. 3TC, one of the NRTIs, is a newly discovered P2X7 receptor inhibitor, which attenuates the progression of neuronal and vascular lesions in DR. Treatment with 3TC reduces the number of cell-free capillaries in the retinas of diabetic mice [43]. We used A740003 and MCC950, the specific inhibitors of P2X7 and NLRP3, respectively, to preprocess the cells. The protein expression levels of P2X7 and NLRP3 remarkably decreased after A740003, 3TC, and MCC950 treatments (Fig. 7a). Apoptotic and pyroptosis rates also decreased (Fig. 7b, d). Immunofluorescence of cells stimulated with a combination of HG and LPS for 7 days shows that the cells co-expressed P2X7 and NLRP3 (Fig. 7c). The three inhibitions attenuated the upregulation of the secretion of inflammatory cytokines, including IL-6, ICAM-1, TNF-α, and IL-1β, in cell supernatants (Fig. 7e).

## Discussion

An increasing body of evidence shows that an ongoing sub-clinical, low-grade inflammation is closely involved in the pathogenesis of type 2 diabetes and its associated complications. In diabetes, many pathogenic bacteria and their metabolites can enter the circulation via a breach in the intestinal barrier, resulting in systemic effects, including chronic low-grade inflammation, dysregulated lipid metabolism, and insulin resistance [44, 45]. Multiple stressors trigger REC apoptosis, and the most studied among which are high glucose levels and inflammatory factors. Increased in LPS level is an underlying trigger for low-grade inflammation and stress signaling, often observed in type 2 diabetes [37]. The CARD domain of caspase 11 can recognize LPS to form the oligomerization complex, which is then activated. After activation, caspase 11 shears the GSDMD carboxylterminal and creates a 30 kDa amino-terminal (GSDMD-NT) active fragment [20, 46]. After oligomerization, multiple GSDMD-NT segments migrated to the cell membrane, forming stable annular pores with inner and outer diameters of 15 and 32 nm, respectively, on the cell membrane, leading to cell pyroptosis [47, 48]. In addition, LPS stimulation induces caspase-11-dependent cleavage of the pannexin-1 channel and ATP release, which in turn activates the purinergic P2X7 receptor to mediate cytotoxicity [21]. Activated caspase-11 activates the NLRP3/ASC-Casp-1 pathway, enabling cells to secrete the pro-inflammatory factors IL-1β and IL-18 [49]. Furthermore, pannexin1 and P2X7 are required for susceptibility to endotoxic shock induced via the noncanonical inflammasome pathway [21].

**Fig. 8:**
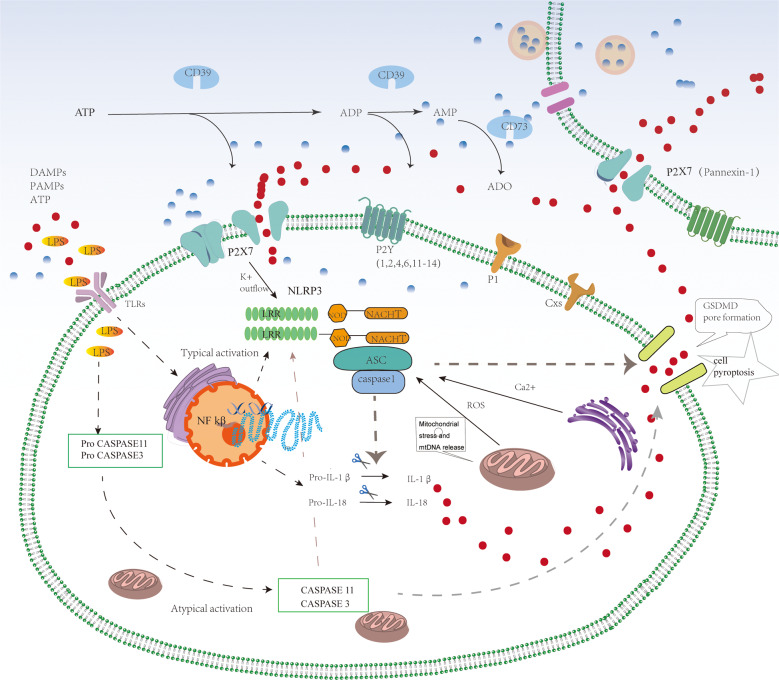
In DR, increased levels of LPS activates the inflammasome in typical and atypical forms. The atypical inflammasome pathway activates caspase-11, which directly causes pyroptosis. In a specific model, NLRP3 causes procaspase-1 to be cleaved into active caspase-1, and activated caspase-1 cleaves IL-18 and IL-1β, resulting in cell pyroptosis. Simultaneously, P2X7 activation, cell death and the release of many inflammatory factors significantly increase the secretion of ATP, forming an inflammatory cascade, and finally, the formation of P2X7 macropores, leading to cell death.

ATP might be the most ancient extracellular messenger used by primordial cells to send messages to their neighboring cells or as a passive signal of danger or distress [50]. ATP and other nucleotides exhibit all the fundamental features of bona fide extracellular messengers. First, they are present in small amounts (nmol/l) in the extracellular space under physiological conditions, such as resting cells or healthy tissues. Second, high amounts of ATP are stored intracellularly (from 5 to 10 µmol/l) [51]. Extracellular ATP may act as a “danger” signal ubder pathological conditions. Almost all cells release ATP in the extracellular environment under specific stimulation [52]. ATP can be released after cell damage and death or from living cells via different channels. The release mechanisms include secretory exocytosis, connexin or pannexin hemichannels, ATP binding cassette (ABC) transporters, calcium homeostasis modulator channels, ATP-gated P2X7R, and two classes of channels maxi-anion channels, and volume-regulated ion channels [53]. We hypothesized that in RECs cultured under HG condition, LPS activated the P2X7 macropore opening via an autocrine ATP positive feedback loop, which then activated the NLRP3 inflammasome, amplified the inflammatory response, and promoted cell death. Activating the NLRP3 inflammasome amplifies the inflammatory response and promotes cellular death. Simultaneous activation of the caspase-11 and caspase-1 pathways induces NLRP3-dependent pyroptosis (Fig. 8). ATP is a potent inducer of NLRP3 activation and IL-1β maturation and is released simultaneously [54]. Under conditions of short ATP, the cation channel of the P2X7 receptor opens, leading to K^+^ outflow and Na^+^ and Ca^2+^ inflow. Under conditions of continuous ATP stimulation, P2X7 receptors form non-selective membrane pores, allowing some substances with a relative molecular mass of 900 kDa to enter the cell, leading to cell death [42]. Dysregulation of the overactive P2X7 calcium signal dysregulation is critical for the death of neurons and microvascular cells [55].

Activation of P2X7 signaling due to high glucose levels, disrupts the blood-retinal barrier and promotes the release of IL-1β in hRECs [56]. Müller cells in RNUT induce programmed REC death via a dual mechanism of ATP release and P2X7 upregulation, which may be necessary for vascular injury in DR [11]. IL-1β is essential in the development of retinopathy in diabetes [57]. The pannexin-1 channel is activated by cleavage o its COOH-terminal autoinhibitory domain by caspase-3 in response to apoptotic stimuli or by purified caspase-3 [58–60]. We observed the upregulation of caspase-3 after LPS induction in the HG medium in this study.

Additionally, we provided evidence for the precise mechanism underlying mREC apoptosis after exposure to LPS and HG. Our results revealed that P2X7 expression increased in mRECs treated with a combination of HG and LPS and in STZ-induced mice with DR. The upregulation of P2X7 expression aggravated DR in vitro and vivo. This trend is consistent with the results of previous studies showing that P2X7 levels increased in endothelial cells and in the retinas under diabetes conditions [21, 61]. Conflicting results have been obtained in vitro studies on HG-induced RECs. We have observed that short-term hyperglycemia did not increased REC apoptosis or promoted cell proliferation. However, mRECs cultured with HG showed significantly higher apoptotic rates and cell deaths when stimulated with LPS. This indicates that the increase in P2X7 expression in cells under HG condition decreases the stimulation threshold of LPS with respect to cell death, and that LPS simultaneously forms an ATP autocrine feedback loop by activating P2X7, initially resulting in ATP release and triggering the assembly of the NLRP3 inflammasome, followed by continued release, amplification and perpetuation of the inflammasome pathwayand induction of pyroptosis.

3TC reversed this detrimental effect. We treated mRECs and DR mice with A740003 and MCC950, the specific inhibitors of P2X7 and NLRP3, respectively. We observed inhibition of the P2X7/NLRP3 inflammatory signaling pathway in the 3TC-treated group, similar that observed in the A740003-treated group; furthermore, 3TC significantly reduced the endothelial cell apoptotic rate. Treatment with 3TC or A740003 reversed the detrimental effects of HG and LPS. This highlighted the involvement of the P2X7/NLRP3 pathway in the pathogenesis of diabetic retinal dysfunction and the therapeutic potential of 3TC in ameliorating diabetic endothelial damage.

Furthermore, we confirmed that stimulation of mRECs with LPS induced the release of IL-1β, which was inhibited by MCC950. Cytolysis is mediated by opening of the P2X7-associated pore in response to nM concentrations of ATP. The activity of the P2X7 pore that mediates influx or efflux of large molecules has been linked to cytolytic activity. It is distinct from that of the P2X7 channel that induces K^+^ efflux, suggesting that P2X7 is required for organelle damage and cytolysis, triggered by the opening of the P2X7-associated pore [11]. Pannexin 1 mediates the release of ATP. PANX1 activation during apoptosis requires caspase-mediated cleavage of PANX1 at its C-terminus. High extracellular K levels activate PANX1 in rat neurons and astrocytes as part of the inflammasome [62]. The cells can release ATP in different ways, including membrane stress or damage and molecular (Panx1, Connexin, P2X7) and vesicular transport. Once on the extracellular side of the membrane, ATP either follows a degradative pathway or stimulates P2 receptors [63]. Under normal conditions, cytosolic concentrations of ATP range from 3 mM to 10 mM, whereas the amount of extracellular ATP vary widely from picomoles to micromoles with cells and tissue types [64]. Injured cells can release ATP through the cytomembrane in different ways. ABC transporters, such as cystic fibrosis transmembrane regulator, regulate the release of ATP via ion channels [65]. Gap junctions, membrane channels, hemichannels such as connexin and pannexin, and P2 receptors are all involved in ATP release.

ATP release can also occur via P2X7R, especially when this receptor is over-stimulated and the associated large conductance pore (the macropore) is completely opened, thereby allowing the transit of molecules up to 900 kDa in size [66]. In addition, P2X7R plays a unique role in inflammation, as its stimulation activates the NLRP3 inflammasome and the maturation and secretion of IL-1β [16]. Most P2X7-dependent pro-inflammatory responses, including ATP release, are due to the opening of macropore. Macropore is intrinsic to the P2X7R [21, 67]^.^ P2XRs, notably P2X7R, are involved in the pathogenesis of diabetes because of their ability to trigger inflammasome activation and release of inflammatory cytokines [13].

Our study has a limitation. We did not elucidate the molecular basis of conversion of P2X7 from an ion channel to a pore, which remains unclear. Genetic experiments showed that P2X7, and not pannexin-1, is required for P2X7-associated pore formation induced by ATP stimulation. K^+^ efflux is widely accepted as a standard upstream signal for NLRP3 inflammasome activation [68]. A recent study demonstrated that ATP-induced activation of the P2X7 receptor and NLRP3 inflammasome was promoted by paxillin, and that the extracellular ATP-induced phosphorylation of paxillin led to the formation of the P2X7R-Paxillin-NLRP3 complex. The complex construction then facilitated NLRP3 inflammasome complex assembly via deubiquitination of NLRP3 [39]. ATP exposure stimulates glutathione efflux, which is a necessary switch for NLRP3 inflammasome activation [40]. This mechanism plays a significant role in activating NLRP3 inflammatory vesicles and warrants further investigation.

In conclusion, hyperglycemia can increase the cellular damage caused by LPS, which included pro-inflammatory, pro-apoptotic, and pro-pyroptosis events. Inhibition or deletion of the P2X7 receptor in LPS-primed macrophages attenuated cytokine production and inflammatory signaling. 3TC attenuated LPS-induced pro-inflammatory, pro-apoptotic, and pro-pyroptosis effects under hyperglycemia by targeting the P2X7/NLRP3 pathway, preventing tissue damage, cell apoptosis, cytokine production, and inflammatory signaling pathway activation.

## Materials and methods

### Experimental animals

Eighty male C57BL/6J mice (weighing 18–22 g, aged 6 weeks) were obtained from the Experimental Animal Center at the Qilu Hospital of Shandong University. After stable feeding, the mice were randomly divided into control (Group 1, *n* = 15) and diabetic groups(Group 2, *n* = 60). Animals in the diabetic group were established by intraperitoneally administering 50 mg/kg STZ (Sigma, St. Louis, MO, USA; freshly diluted in 0.1% mol/l citrate buffer, pH 4.5) daily for 5d. The same volume of citrate buffer without STZ was used for a control group. I list all the mice parameters, including body weight and blood glucose(Supplementary Table S1).The model was considered successfully established if blood glucose >16.7 mmol/L. After the successful establishment of the diabetic model, the diabetic group was randomly divided into four subgroups containing an approximately equal number of animals (*n* = 13–15), including the A740003 group (100 µg/kg/d, Group A), MCC950 group (100 ug/kg/d, Group B), 3TC Group (100 ug/kg/d, Group C) and a diabetic group treated with the same dose of normal saline (Group D). A74003 (GC11842, GLPBIO, CA, USA) was injected into Group A mice every alternate day for the last 4 weeks before the end of the experiment. MCC950 (GC31644, GLPBIO, CA, USA) was injected into Group B mice for 3 consecutive days after the first dose was administered, then every alternate day for the last 4 weeks. 3TC (GC10310, GLPBIO, CA, USA) was injected into Group C mice for 5 consecutive days per week for the last 4 weeks(Supplementary Fig. S1). The retinas were obtained after 4 and 12 weeks of STZ treatment, and the samples were fixed or stored at specific temperatures for subsequent imaging or biochemical analyses.

The study was approved by the Ethics Committee of The Qilu Hospital of Shandong University. All animal studies and experimental protocols were carried out in accordance with the Animal Management Rule of the Ministry of Health in the People’s Republic of China (Document No. 55, 2001) and the Guide for the Care and Use of Laboratory Animals published by the US National Institutes of Health (NIH Publication No. 85-23, revised 1996).

### Cell culture

mRECs were obtained from the Qingqi Company (Shanghai, China), cultured in Dulbecco’s modified Eagle’s medium (HyClone; GE Healthcare Life Sciences, Logan, UT, USA) supplemented with 10% fetal bovine serum (Sciencell, San Diego, CA, United States), and incubated in a humidified atmosphere of 5% CO_2_ at 37 °C. The cells (1 × 10^5^) were cultured in per well of six-well plates. mRECs at passages 3–6 were used in the experiments.

### In vitro stimulation

The experiments were conducted in three stages. In the first phase, the cells were pretreated with different glucose concentrations, separately 5.5 mM D-glucose, 30 mM D-glucose, 50 mM D-glucose, In the second phase, the cells were treated with LPS (10 ng/ml, L2880, SigmaAldrich, St. Louis, MO, USA), BzATP (100 µmol/l, 112898-15-4, SigmaAldrich), TNF-α (10 ng/ml, 315-01 A, PeproTech) either alone or with HG (30 mM D-glucose) or NC (5.5 mM D-glucose) for 48 h. In the third phase, endothelial cells were incubated with an HG medium(30 mM D-glucose). We added A740003 (100 µmol/ml), MCC950 (100 mol/ml), and 3TC (100 µmol/ml) were added to cells 6 h prior to stimulation with LPS and incubated for 72 h.

### RNA isolation and cDNA synthesis

At the end of the each time point, isolated total RNA using Trizol reagent (Life Technologies, Grand Island, NY, USA). RNA was quantified using 260/280 UV spectrophotometry. Total RNA pellets were resuspended in RNase-free water, followed by removal of potentially contaminated DNA by treatment with DNase I (Life Technologies). Next, 1 μg of total RNA from each sample was used for reverse transcription with a ReverTra Ace qPCR RT Kit (TOYOBO, Japan) to generate first-strand cDNA in a 20 μl reaction mixture. Finally, the cDNA was stored at −20 °C before use.

### Quantitative reverse transcription polymerase chain reaction (qRT-PCR)

qRT-PCR was performed to measure mRNA expression with the following primers (Supplementary Table S2). qRT-PCR was performed with SYBR® Green Real-time PCR Master Mix (TOYOBO, Japan), The cycling conditions were as follows: denaturation step at 95 °C for 30 min followed by 40 cycles of standard PCR.The specificity of the amplified products was determined by melting curve analysis. Quantification was performed with the 2-ΔΔCt method. Gene expression values were normalized agains GAPDH mRNA.expression, which proved to be stable across the samples.

### Detection of cytokines in the culture medium

IL-1β、IL-6、TNF-α, and ICAM-1 released by mRECs was measured using a commercially available ELISA kit (R&D System, Minneapolis, MN, USA) according to the protocol described by the manufacturer. ELISA was conducted on culture media collected after treatment. Media samples were immediately centrifuged for 5 min at 4000 × *g* to collect conditioned culture supernatant, stored at − 80 °C until use.

### Measurement of extracellular ATP and plasma LPS

Cells were prepared for ATP release assays. The ectoATPase inhibitor β, γ-methylene-ATP (300 lM; Sigma-Aldrich Corp) was added 15 min prior to each stimulation. Extracellular ATP in mRECs culture medium was measured 15 min and 45 min after LPS stimulation using an ATP bioluminescence assay kit (Sigma-Aldrich Corp). Luminescence was quantifified using a luminometer (TD 20/20; Turner Designs, San Jose, CA, USA). Concentrations of ATP were calculated using an ATP standard curve.

Evaluation of LPS in peripheral blood supernatant was a quantitative test for Gram-negative bacterial endotoxin (Lonza, USA; Cat No. QCL-1000).

### Western blot analysis

Protein was harvested from mice retinal tissue and mRECs using RIPA buffer with protease inhibitors, and the protein concentration was determined by the BCA protein assay (Beyotime, China). Protein samples were separated with 10 and 12% SDS–PAGE in a running buffer and transferred to PVDF membranes with a transfer buffer. After blocking in 5% skim milk, the membranes were incubated with the primary antibodies,followed by incubation with secondary antibody for 1 h at room temperature.Antibody sources and dilutions are shown in Supplementary Table S3. After rinsing, the membranes were transferred into the Amersham Imager 600 system and covered with 200 μL of BeyoECL Star (Beyotime,Biotechnology, China).The immunoreactive complexes were captured with a fluorescent imager (Amersham Imager 600 RGB, GE) and Bio-Rad Image Lab software for quantifification. All experiments were repeated at least three times.

### Flow cytometry analysis of cell apoptosis

FITC-conjugated Annexin V (Annexin V-FITC)/propidium iodide (PI) Apoptosis Detection kit was purchased from Beyotime Biotechnology (Shanghai, China). After treatment and centrifugation at 2000 rpm for 5 min under the condition of 4 °C, mRECs were harvested and added with binding buffer to re-suspend, followed by staining in the dark with Annexin V-FITC and PI for 15 min. A FACSCalibur flow cytometer (BD Biosciences, Franklin Lakes, NJ, United States) was employed to detect and analyze the apoptosis of mRECs. The experiments were repeated three times.

### Retinal trypsin digestion assay

The eyes were enucleated and fixed in 4% PFA for 24 h. They were then equatorially bisected, and the retinas were removed. The retinas were incubated with 3% trypsin at 37 °C for 3 h. After repeated washing, the network of vessels was isolated and mounted on slides. The dried retinal vasculature was then stained with periodic acid-Schiff and hematoxylin. Take Images through an inverted light microscope (DMi8, Leica).

### Immunofluorescence

The eyeballs were fixed with 4% PFA and embedded in paraffin to generate retinal cross-sections (10 µm). The paraffin sections were first dewaxed. After heat-mediated antigen retrieval with citrate buffer, the sections were permeabilized with 0.2% Triton X-100 for 30 min and blocked with 5% FBS for 2 h at room temperature. The mREC cell cultures were fixed in the Petri dishes with 4% paraformaldehyde in PBS for 30 min. Permeabilized with 0.1% Triton X-100 in 0.1% sodium citrate on ice for 2 min. The cells were washed thrice with PBS for 5 min each wash. Then, the samples were incubated with a primary antibody at 4 °C overnight. The following day, the samples were incubated with secondary antibody (Supplementary Table S3) for 1 h, followed by DAPI (1:1000) counterstaining for 10 min at room temperature.

Fluorescent images were taken using a multispectral panoramic tissue scanning microscope (TissueFAXS Spectra, Zeiss). (Leica, Germany). The preparations were evaluated independently by two observers,the observers were blinded to each other’s findings until every observer had finished their evaluations.

### Terminal deoxynucleotidyl transferase dUTP nick end labeling (TUNEL) analysis

After incubation with HG and LPS, the mREC cell cultures were fixed in the Petri dishes with 4% PFA in PBS for 30 min and then permeabilized with 0.1% Triton X-100 in 0.1% sodium citrate on ice for 2 min. The cells were washed three times in PBS for 5 min each wash. The samples were stained using a TUNEL BrightGreen Apoptosis Detection Kit (Vazyme, A112-01).

### Evans Blue (EB) permeation assay

The mice were anesthetized with ketamine (80 mg/kg) and xylazine (4 mg/kg). Evans blue (EB, 20 mg/ml in saline; Solarbio, China) was injected through the tail vein at a dose of 45 mg/kg. After the dye had circulated for 2 h, the mice were euthanized with high doses of ketamine and xylazine, and the eyes were enucleated and immersed in fresh 4% PFA for 30 min. Then, whole retinal mounts were prepared, and EB leakage was examined by laser confocal microscopy (LSM880, Zeiss).

### Statistical analyses

All data are expressed as means ± SD,statistical analyses were carried out using GraphPad Prism 8.0 (GraphPad Software Inc. La Jolla, CA, USA) statistical analysis of more than two groups. *p* < 0.05 was considered statistically significant. One-way ANOVA or a Student’s *t* test was performed to assess statistical differences, *p* < 0.05 was accepted as statistically significant.

## Supplementary information

supplementary materials

## Data Availability

The data used to support the findings of this study are available from the corresponding author upon request.
