# Correction: Targeted P2X7/NLRP3 signaling pathway against inflammation, apoptosis, and pyroptosis of retinal endothelial cells in diabetic retinopathy

**DOI:** 10.1038/s41419-025-07682-1

**Published:** 2025-05-16

**Authors:** Hui Kong, Hongran Zhao, Tianran Chen, Yanling Song, Yan Cui

**Affiliations:** 1 Shandong University of Traditional Chinese Medicine, Jinan, Shandong Province China; 2 Department of Ophthalmology, Qianfoshan Hospital of Shandong First Medical University, Jinan, Shandong Province China; 3 NHC Key Laboratory of Otorhinolaryngology, Qilu Hospital of Shandong University, Jinan, Shandong Province China; 4 Department of Ophthalmology, Qilu Hospital of Shandong University, Shandong University, Jinan, Shandong Province China; 5 Shandong University, Jinan, Shandong Province China

Correction to: *Cell Death and Disease* 10.1038/s41419-022-04786-w, published online 12 April 2022

In Figure 2b, the images for the retinal flat mounts of the DR group and NC group were saved in different folders, and an incorrect image was selected during the completion of the figures.
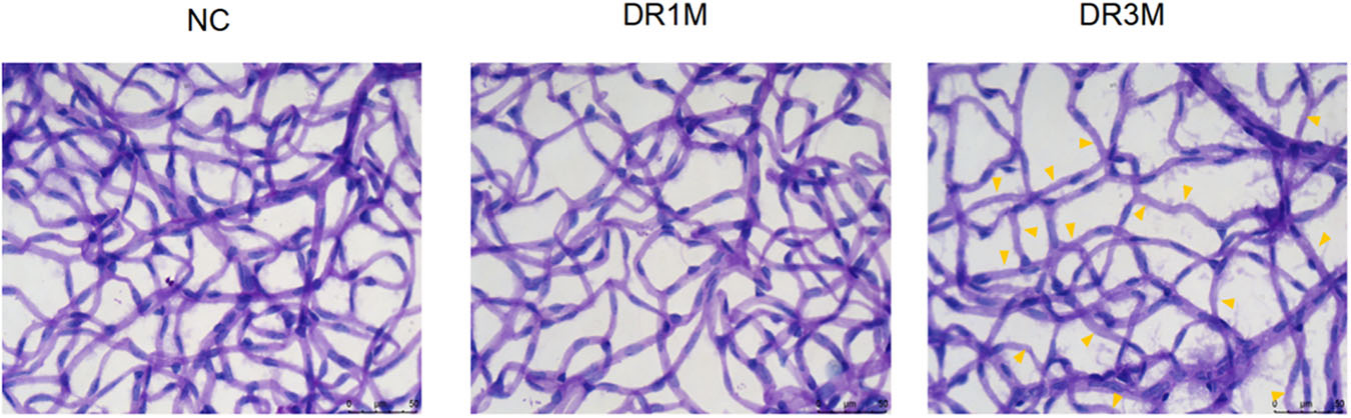


In Figure 3C, The scatter plot of TNF-α is incorrect. The correct figure has been verified and presented, which does not affect the results in the text.
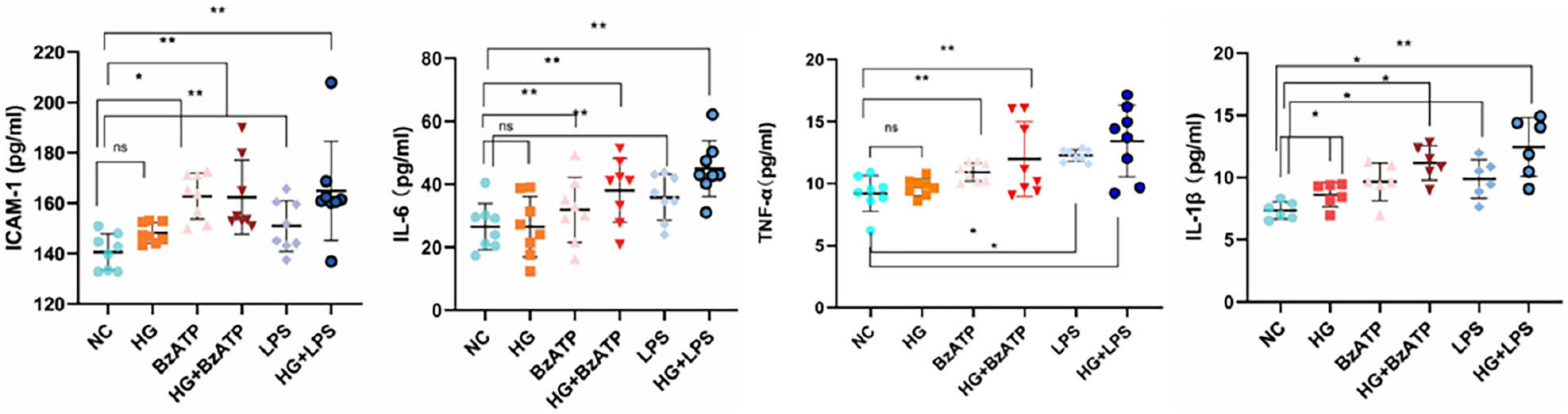


Figure 5a presents a similar issue as 2b; during the submitting process, the Western Blot images underwent complex grouping, leading to a mix-up in the final use.
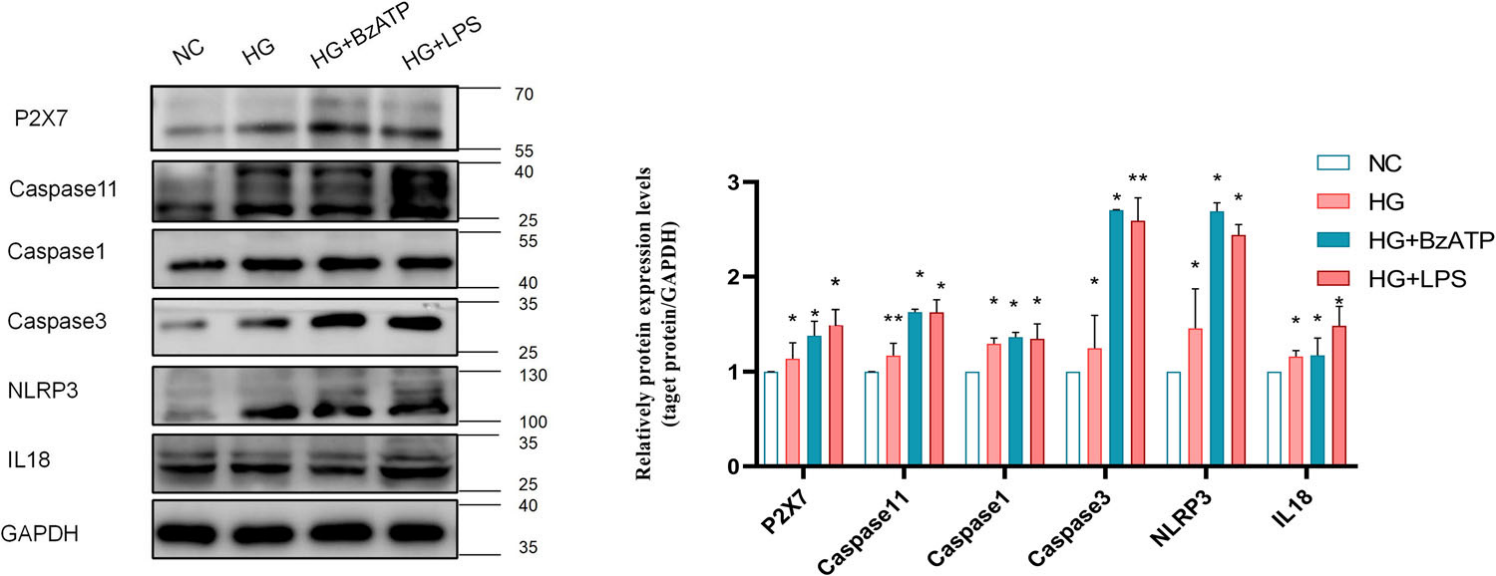


The original article has been corrected

## Supplementary information

original data

