# Supplementary material for: Targeted P2X7/NLRP3 signaling pathway against inflammation, apoptosis, and pyroptosis of retinal endothelial cells in diabetic retinopathy
Source: Cell Death Dis. 2022 Apr 12;13(4):336. doi: 10.1038/s41419-022-04786-w (PMC9001662; doi:10.1038/s41419-022-04786-w)
Supplement: Supplementary file 1 — supplementary materials [file 41419_2022_4786_MOESM1_ESM.docx]

Table s1 General physiological parameters of diabetic and non-diabetic mice

|  | Initial | |  | 1 months | |  | 3 months | |
| --- | --- | --- | --- | --- | --- | --- | --- | --- |
|  | Body Wt  (g) | Glugose  (mmol/L) |  | Body Wt  (g) | Glugose  (mmol/L) |  | Body Wt  (g) | Glugose  (mmol/L) |
| Un-diabetic | 19.56$\pm$1.81 | 7.09$\pm$3.23 |  | 24.38$\pm$6.12 | 7.61$\pm$3.51 |  | 32.28$\pm$0.56 | 6.84$\pm$5.34 |
| Diabetic | 20.76$\pm$5.07 | 6.93$\pm$4.10 |  | 22.32$\pm$3.65* | 24.83$\pm$4.10* |  | 25.82$\pm$3.11** | 23.37$\pm$1.17* |
| Diabetic+A740003 | 19.87$\pm$0.98 | 7.02$\pm$5.11 |  | 22.87$\pm$4.09* | 23.72$\pm$5.44* |  | 26.56$\pm$3.11* | 24.03$\pm$3.29* |
| Diabetic+3TC | 21.04$\pm$3.29 | 8.02$\pm$1.98 |  | 24.03$\pm$2.53* | 22.62$\pm$4.35* |  | 28.74$\pm$3.11* | 21.76$\pm$5.81** |
| Diabetic+Mcc950 | 20.03$\pm$3.11 | 6.81$\pm$4.15 |  | 23.83$\pm$6.03* | 23.14$\pm$1.19* |  | 26.19$\pm$3.11* | 24.12$\pm$2.69* |

Data are shown as mean ±S.E.M. *Indicates a significant difference compared with the age-matched un-diabetic group.


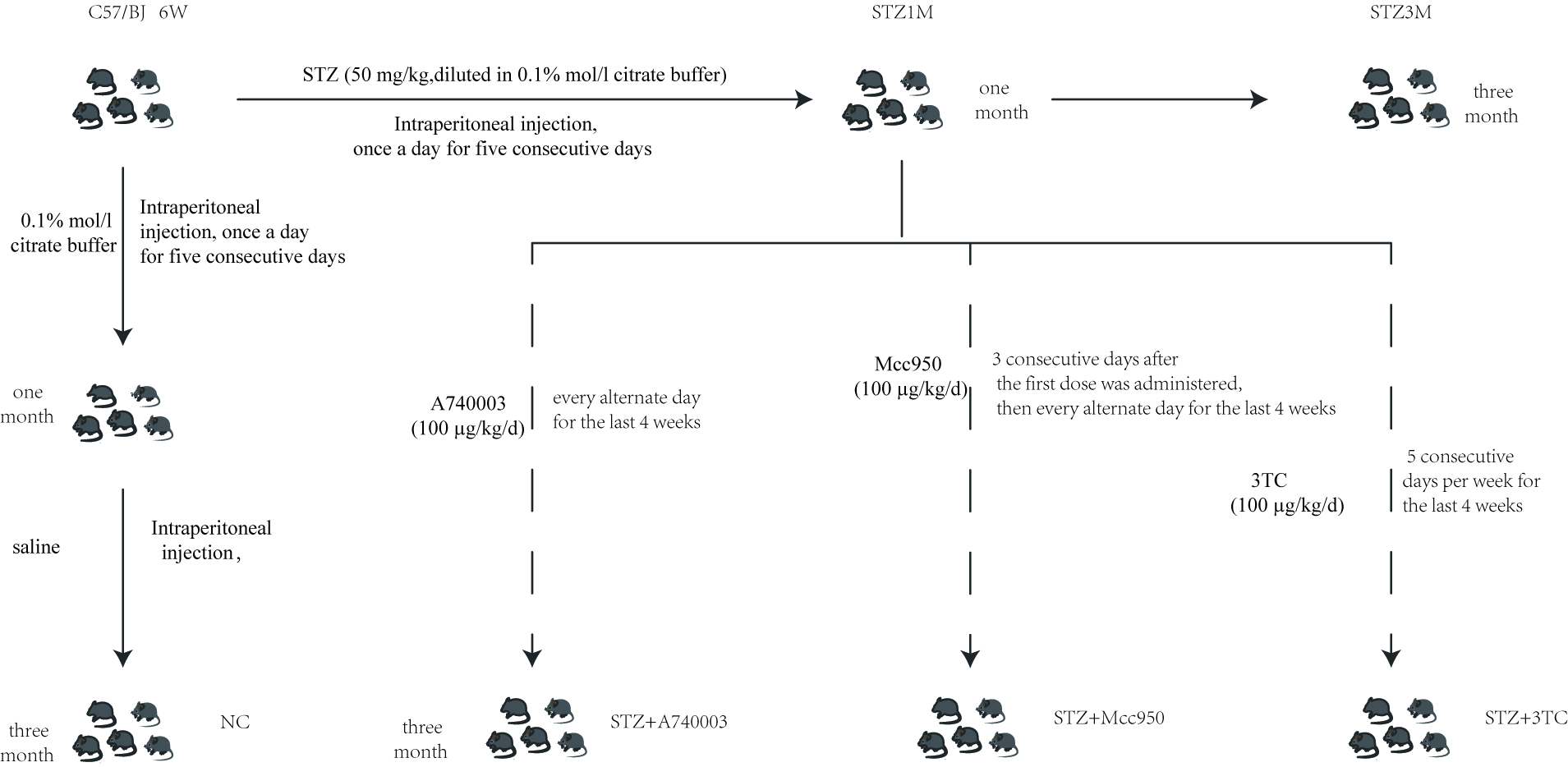


Figure s1 Mouse Injection Process Timeline

Table s2 List of Primer sequences used in this study

| mice | forward | reverse |
| --- | --- | --- |
| P2X7 | 5’-ATACCACGAGAAACTCTTGCC-3’ | 5’-CATGATTCCTCCCTGAACTGC-3’ |
| NLRP3 | 5′-TCTTCTCAAGTCTAAGCACCAAC -3′ | 5′-ACAGCAATCTGATTCCAAAGTC)-3 |
| Capsase1 | 5′TTTCAGGCAAGCCAAATCTTTATCAC-3′ | 5′-TGGTCCAACCCTCGGAGAAAGATG-3′ |
| IL-1β | 5′-TCCTGTCTTGGCCGAGGACTAAGG-3′ | 5′-TC TGGGCTGGACTGTTTCTAATGC-3′ |
| IL-18 | 5′-TGAAGTAAGAGGACTGGCTGTGA -3′ | 5′-TTGGCAAGCAAGAAAGTGTCC-3′ |
| IL-6 | 5′-CATAGCTACCTGGAGTACATGAAGAA -3′ | 5′-GACTCCAGCTTATCTCTTGGTTGA-3′ |
| ASC | 5’-CATCCTGGACGCTCTTGAAAAC -3′ | 5′-CCATAGCCTTCTCGCAGTTGC-3′ |
| TNF-α | 5′-CCCTCACACTCACAAACCACC -3′ | 5′-CTTTGAGATCCATGCCGTTG-3′ |
| Icam1 | 5′-TGTGCCATGCCTTTAGCTCC -3′ | 5′-AAGAGGCTGCCATCACGAG′ |
| Caspase11 | 5’- CAGGGTGGACAAAAGGATTG-3’ | 5’-ACCGTGAAGACAGCTTGACA-3’ |
| GAPDH | 5’-CCTCGTCCCGTAGACAAAATG-3’ | 5’-TGAGGTCAATGAAGGGGTCGT-3’ |

Table 3 List of primary and secondary antibodies used in this study

| Molecular marker | Antibody | Antibody type | Working dilution | Source |
| --- | --- | --- | --- | --- |
| NLRP3 | Rabbit polyclonal | Primary | 1:1000 | Abcam,Cat#ab270449 |
| P2X7 | Mouse monoclonel | Primary | 1:500 | Santa Cruz Biotech,Cat#sc-514962 |
|  | Rabbit polyclonal | Primary | 1:1000 | ABclonal,Cat#A10511 |
| IL-1β | Rabbit polyclonal | Primary | 1:500 | Affinity,Cat#AF5103 |
| IL-18 | Rabbit polyclonal | Primary | 1:1000 | Abcam,Cat#ab191152 |
| Caspase1 | Rabbit polyclonal | Primary | 1:500 | Ptglab,Cat# 22915-1p |
| Caspase3 | Rabbit polyclonal | Primary | 1:2000 | Abcam, Cat #ab184787 |
| Caspase11 | Rabbit polyclonal | Primary | 1:2,000 | Abcam, Cat# Ab246496 |
| GAPDH | Rabbit polyclonal | Primary | 1:5000 | Affinity,Cat#AF7021-BP |
| HRP Conjugated AffiniPure IgG | Goat anti-rabbit | Secondary | 1:10000 | Boster,Cat#BA1054 |
| Dylight 680 | Goat anti-rabbit | Secondary | 1:10000 | Cell Signaling,#5366 |
| Dylight 480 | Goat anti-rabbit | Secondary | 1:1000 | Abbkine,Cat#A23220 |
| Dylight 649 | Goat anti-mouse | Secondary | 1:1000 | Abbkine,Cat#A23640 |
