# Supplementary figures and images for: Correction: Targeted P2X7/NLRP3 signaling pathway against inflammation, apoptosis, and pyroptosis of retinal endothelial cells in diabetic retinopathy
Source: Cell Death Dis. 2025 May 16;16(1):389. doi: 10.1038/s41419-025-07682-1 (PMC12084567; doi:10.1038/s41419-025-07682-1)

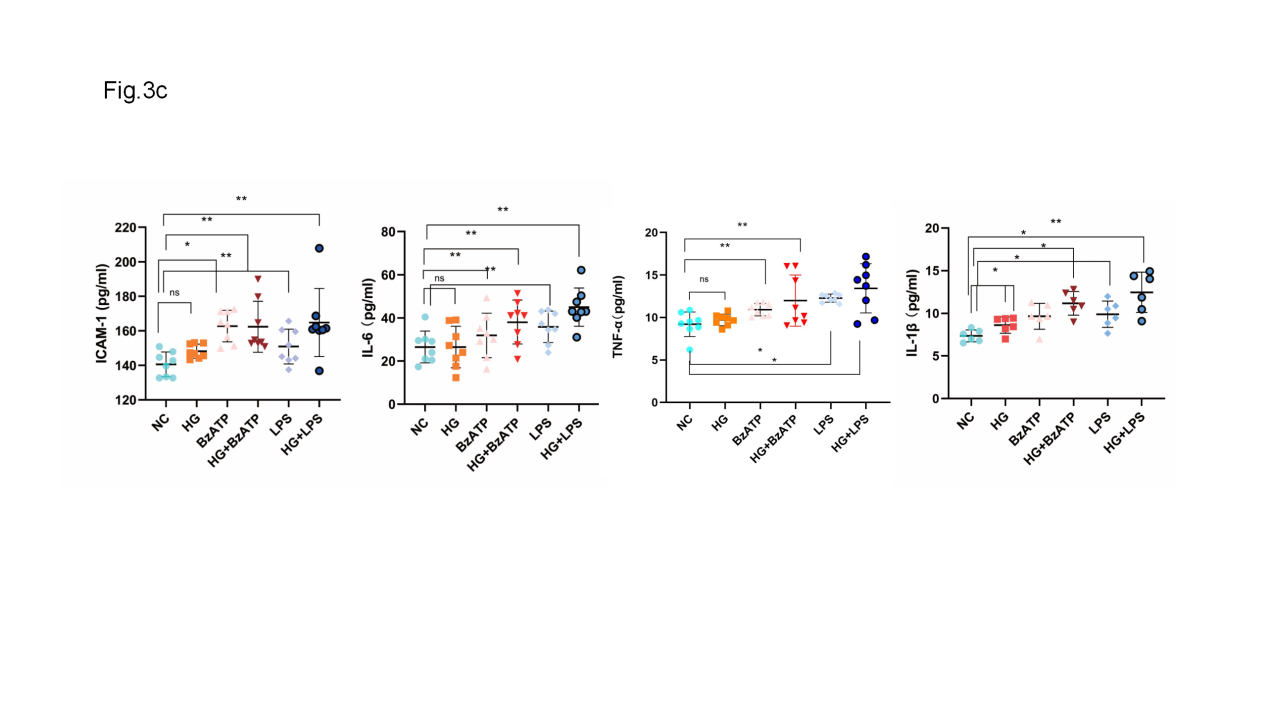

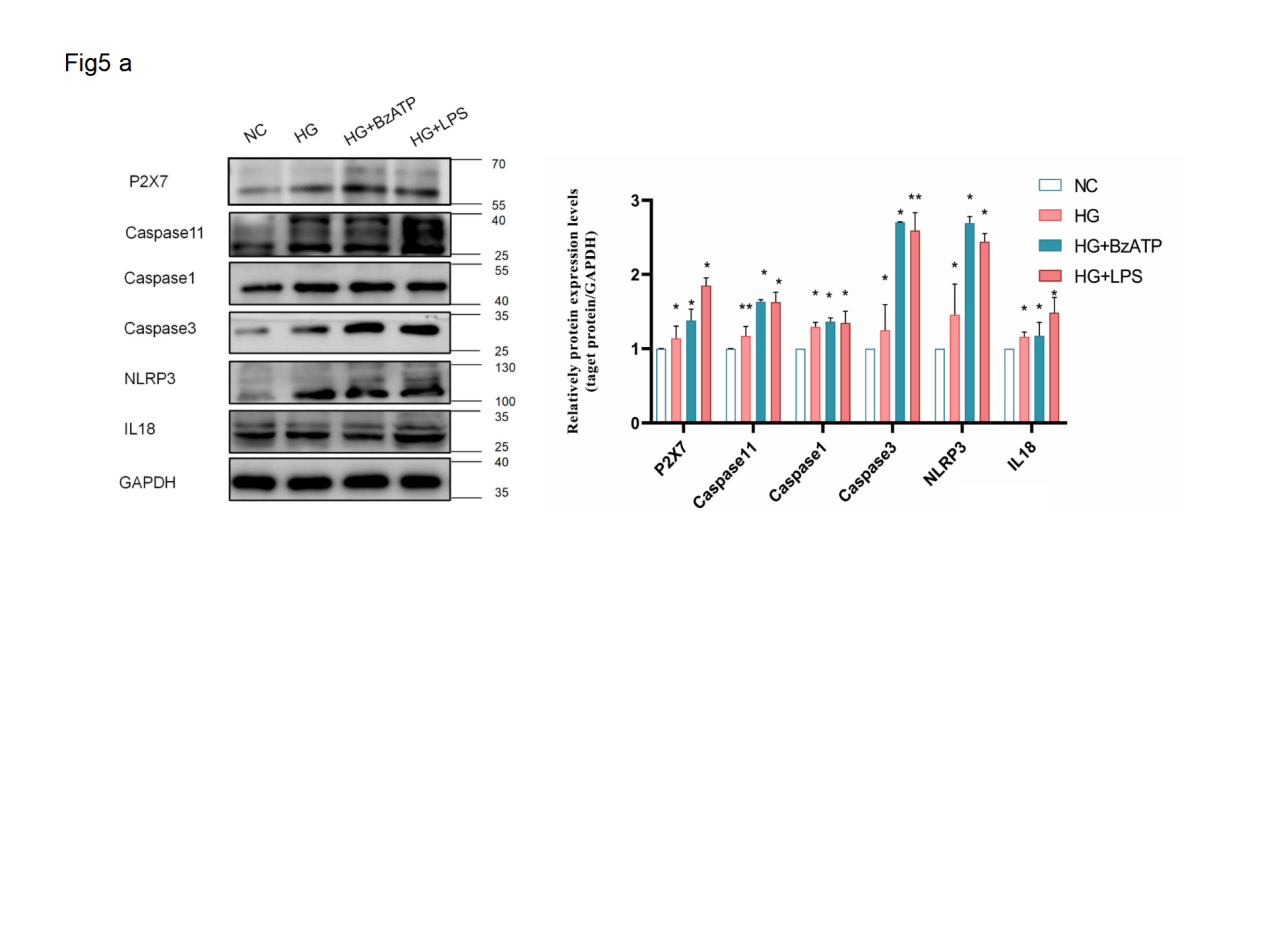

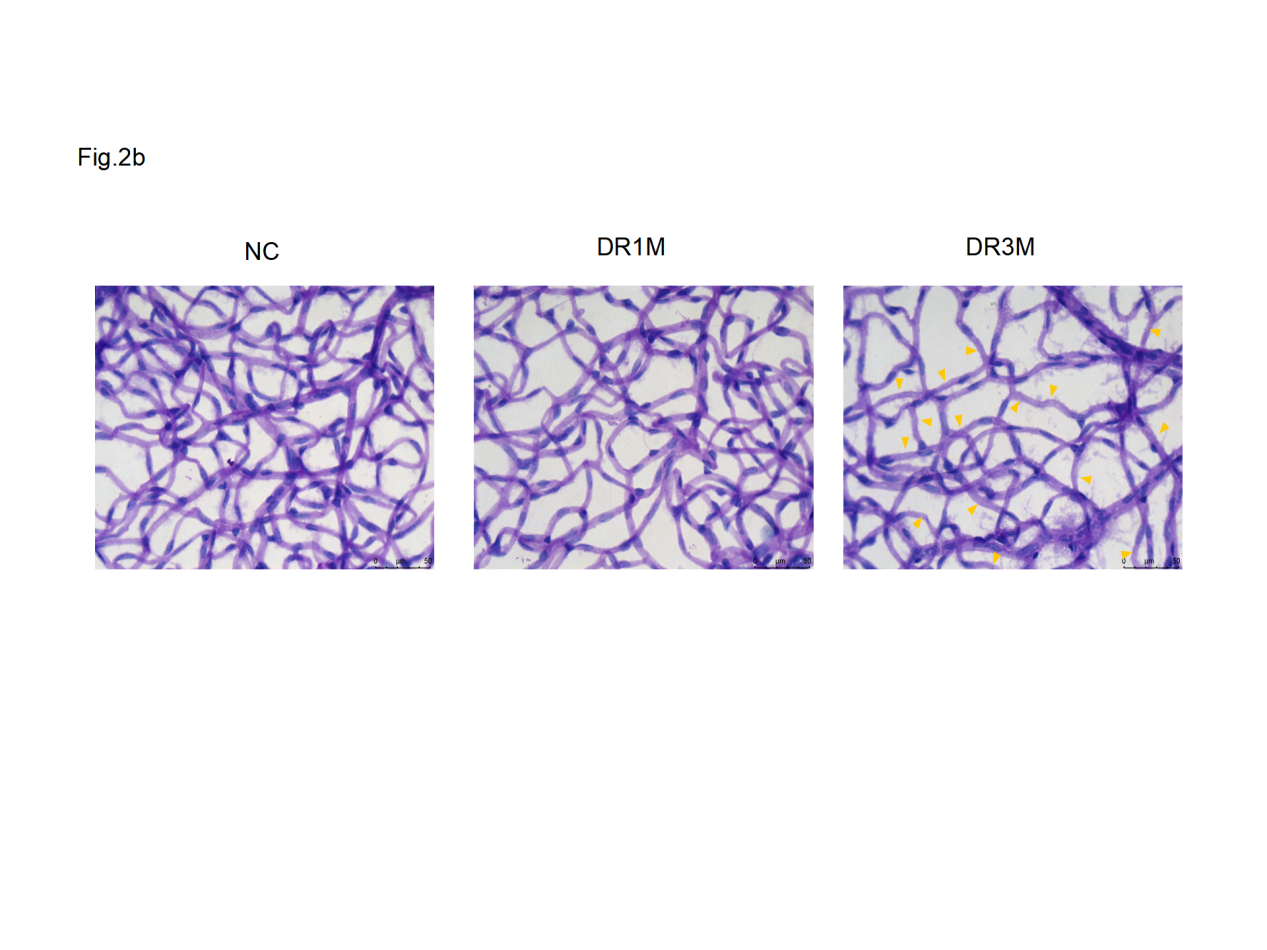


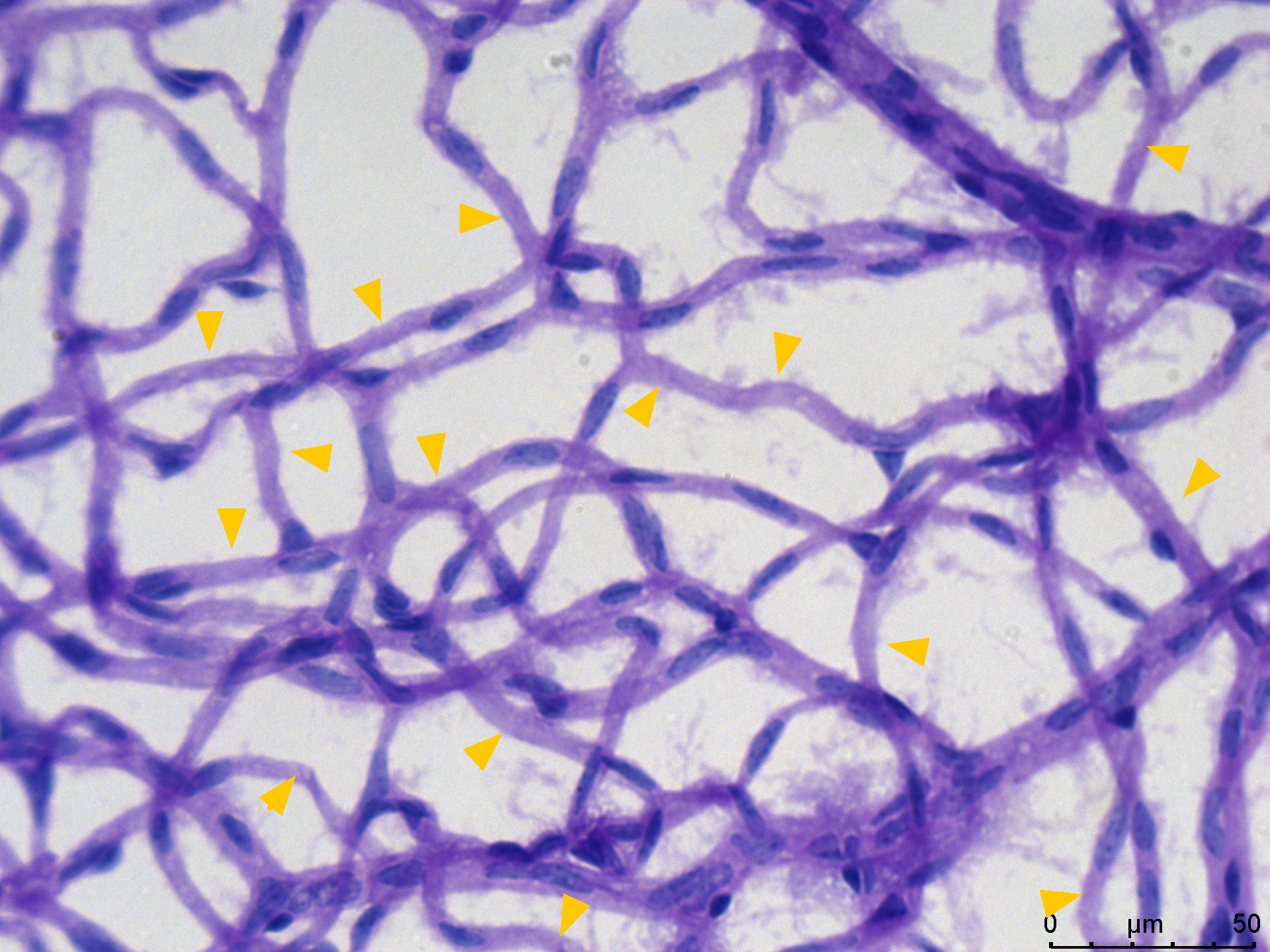


DM1M originaldata


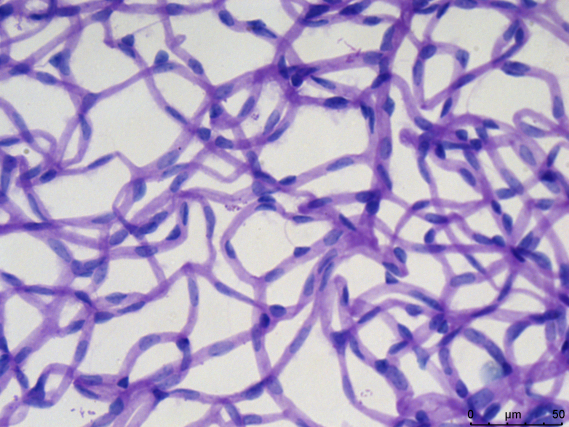


DM1M original data


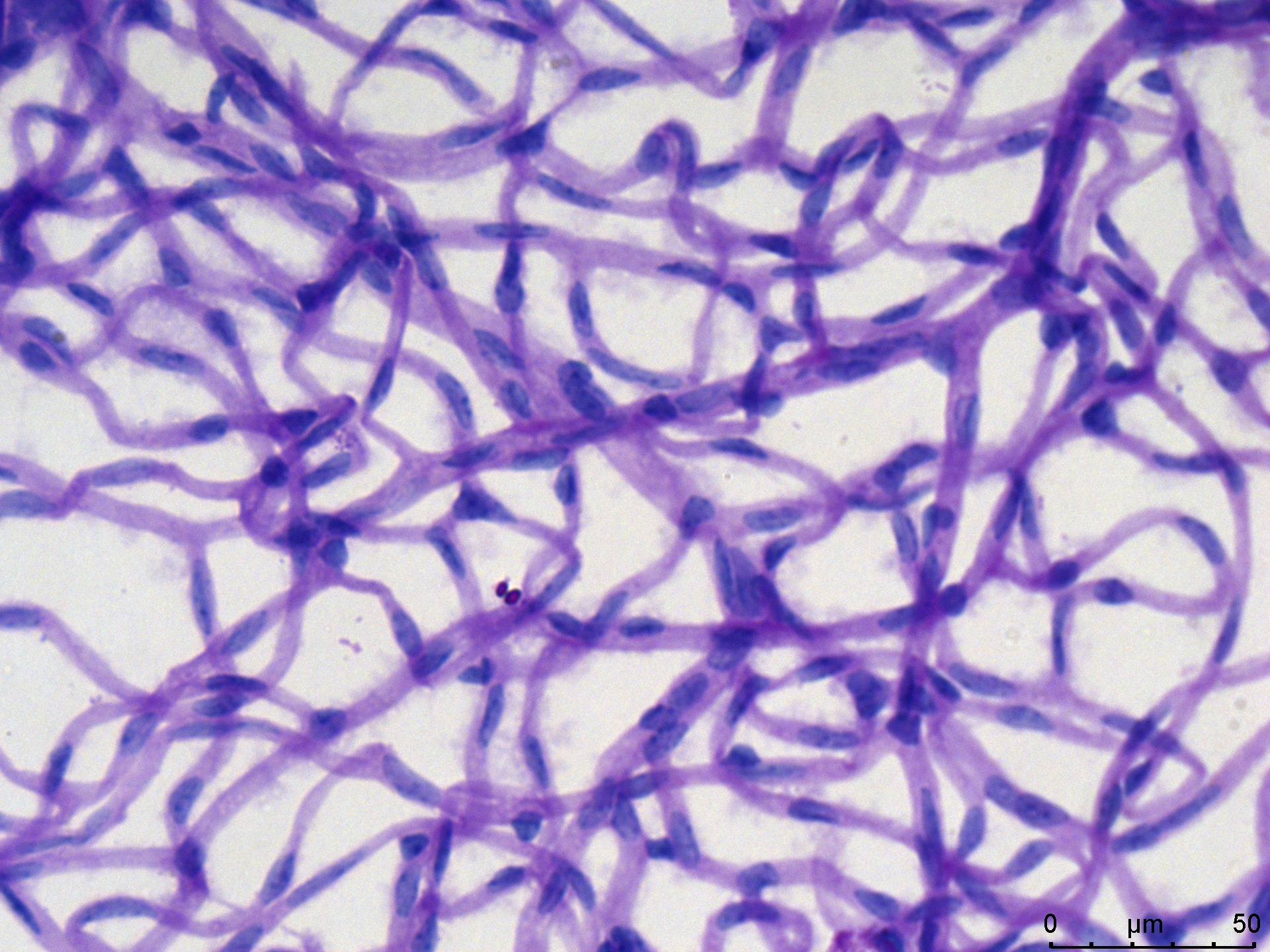


NC original data

P2X7 （WB repeat 3 times）


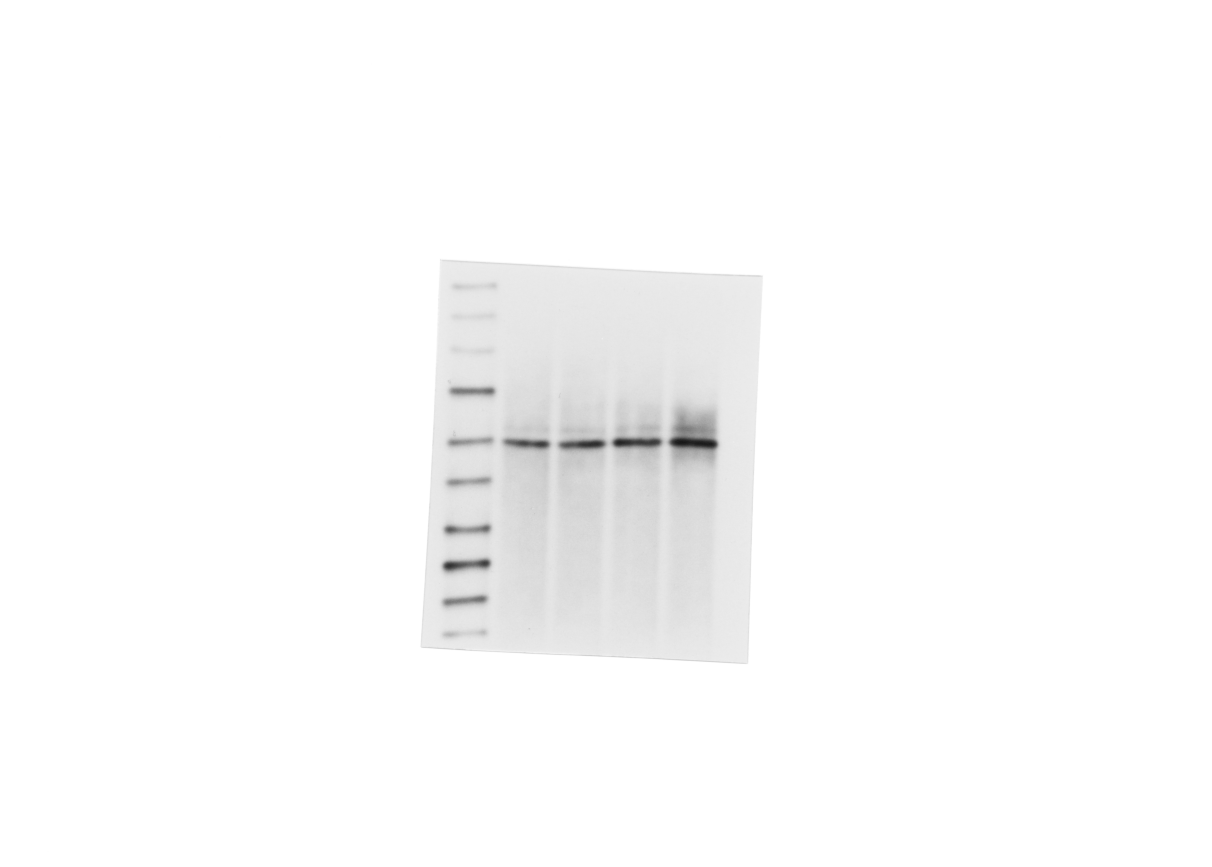

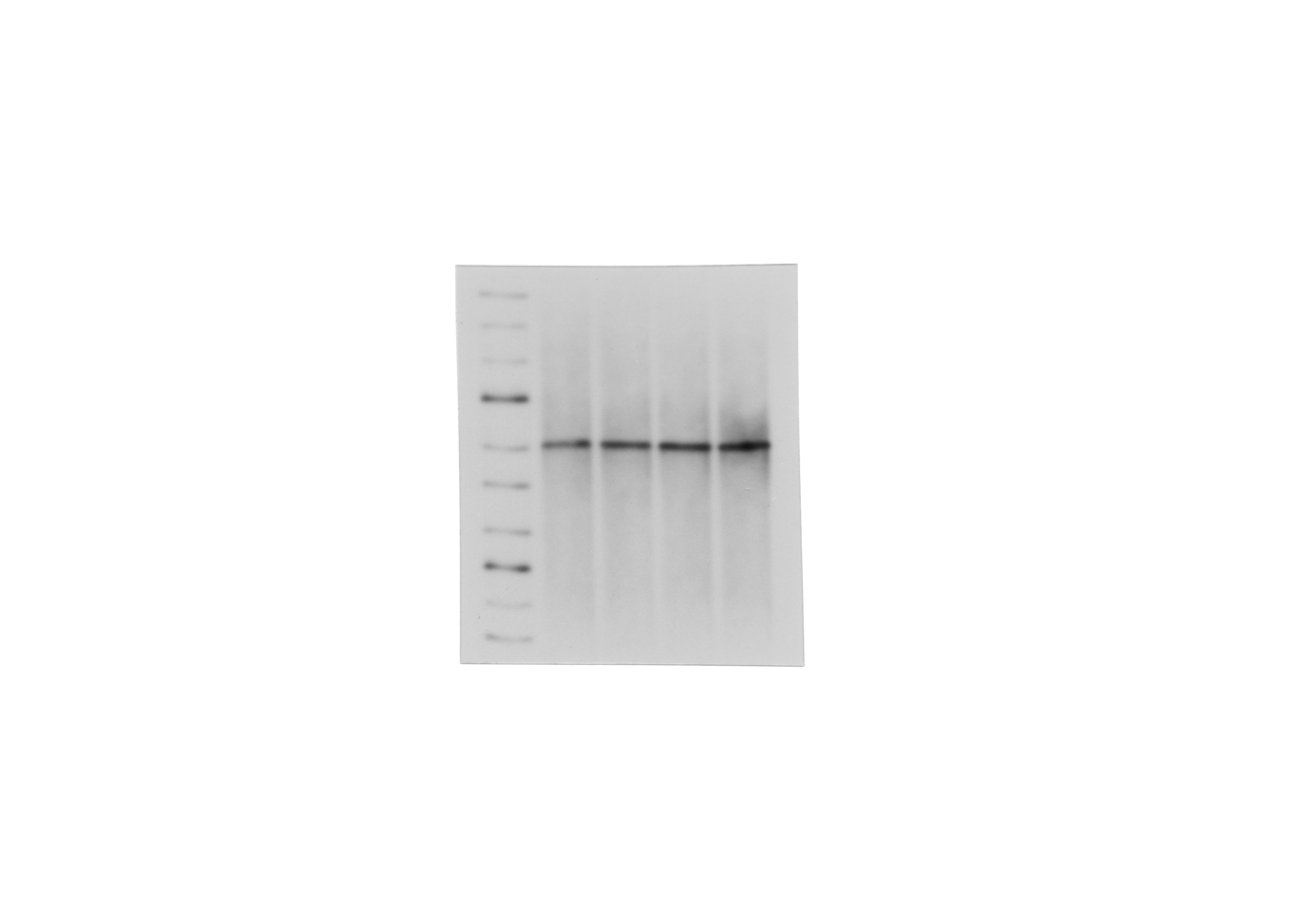

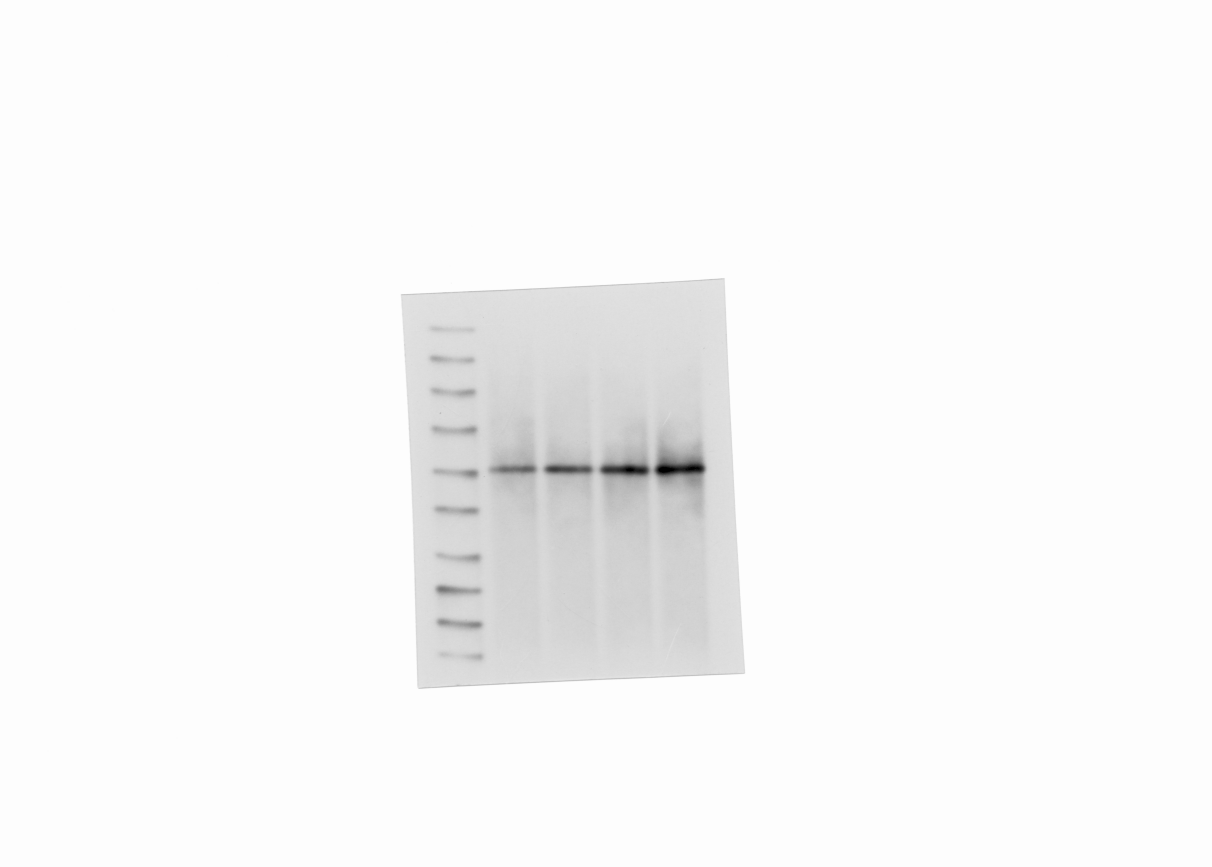

Supplement: Supplementary file 1 — original data [file 41419_2025_7682_MOESM1_ESM.docx]
